# Letter to editor on ‘isolation and characterization of human Wharton's jelly mesenchymal stem cell‐derived extracellular vesicles’

**DOI:** 10.1111/jcmm.70168

**Published:** 2024-10-22

**Authors:** Saravanan Sampoornam Pape Reddy, Delfin Lovelina Francis, Vishal Kulkarni, Sukhbir Singh Chopra

**Affiliations:** ^1^ Department of Periodontology Army Dental Centre (Research & Referral) New Delhi India; ^2^ Saveetha Dental College & Hospitals Saveetha University, SIMATS Chennai India; ^3^ International Agency for Research on Cancer (IARC) World Health Organization (WHO) Lyon France; ^4^ Department of Oral & Maxillofacial Surgery Army Dental Centre (Research & Referral) New Delhi India; ^5^ Commandant, Army Dental Centre (Research & Referral) New Delhi India

Dear Editor,

We have carefully reviewed the methodology described in the study titled ‘Isolation and Characterization of Human Wharton's Jelly Mesenchymal Stem Cell‐Derived Extracellular Vesicles’ by Torabi et al.,^1^ published in the *Journal of Cellular and Molecular Medicine*. This letter concerns the isolation, characterization, and functional assessment of extracellular vesicles (EVs) derived from Wharton's Jelly mesenchymal stem cells (WJ‐MSCs) and their effects on macrophages and stellate cells. While the study presents a comprehensive framework, there are several methodological concerns that warrant further clarification or may pose potential conflicts regarding the robustness and reproducibility of the results. Although the authors provide a comprehensive overview of the isolation and characterization WJ‐MSC derived EVs, the experimental design and interpretation of the data merit further scrutiny.

The authors detail a multistep ultracentrifugation process to isolate EVs, with the final fractions labelled as EV20K and EV110K. However, the reported methods lack adequate controls to confirm the purity of these EV populations. Specifically, there is no mention of assessing the presence of other potential contaminants, such as protein aggregates or apoptotic bodies, which could confound the results. The absence of additional characterization techniques, such as nanoparticle tracking analysis (NTA) or density gradient purification raises concerns about the reliability of the size distribution data obtained via dynamic light scattering (DLS). Furthermore, a more stringent debris removal step, such as a lower‐speed centrifugation or filtration, would enhance the purity of the isolated EV populations.^2^ Given that EV populations differ greatly in size and content, the omission of such steps could influence the downstream biological assays, particularly when assessing functional outcomes.

The authors utilized flow cytometry to measure the uptake of calcein‐labelled EVs by UCB‐derived monocytes. However, the experimental conditions surrounding the labelling and washing steps may inadvertently affect the quantification of EV uptake. It is critical that the authors provide a more detailed methodology regarding how they ensured the complete removal of unbound calcein dye and how this might affect the interpretation of their flow cytometry results. Although Western blotting for EV markers (CD63, CD81) and the absence of calnexin were performed to validate EV identity, it would be beneficial to include NTA in addition to DLS for more accurate size distribution and concentration profiling. The use of calcein AM to label EVs for the uptake study introduces potential issues; calcein dye can leak from vesicles and be taken up by cells, which may inflate the reported uptake rates. A more vesicle‐specific dye (such as DiD or PKH dyes) could reduce this confounding factor, providing a clearer understanding of EV internalization.^3^

The concentration of WJ‐EVs (10 and 50 μg/mL) and the addition of M‐CSF as a positive control are consistent with polarization assays. While the authors included controls (M‐CSF and WJ‐EV‐depleted media), it would strengthen their findings to discuss the rationale behind the chosen concentrations and how they correlate with physiological conditions. Furthermore, the cytokine profile evaluated could benefit from additional inflammatory mediators to paint a more comprehensive picture of macrophage polarization dynamics. However, the inclusion of human platelet lysate (hPL) in the culture medium is a confounding factor, as it can introduce undefined growth factors and EVs from platelets that might have specific effect of WJ‐EVs on monocyte differentiation.^4^

The investigation into the anti‐fibrotic effects of macrophage‐conditioned media on LX2 stellate cells introduces interesting insights. However, the conclusions drawn regarding the therapeutic potential of the EVs should be approached with caution. The authors could have enhanced their discussion by considering the complexities of the in vivo environment, where the interactions among various cell types and soluble factors can differ significantly from in‐vitro findings.

In conclusion, while the study offers valuable insights, addressing the aforementioned concerns could significantly improve the clarity and reproducibility of the results. Further refinement in EV isolation, characterization and culture conditions would help resolve potential methodological conflicts and yield more robust conclusions regarding the biological roles of WJ‐EVs.

Thank you for considering this perspective. We look forward to your response.

## AUTHOR CONTRIBUTIONS

**Saravanan Sampoornam Pape Reddy:** Conceptualization (equal); data curation (equal); formal analysis (equal); writing – original draft (equal). **Delfin Lovelina Francis:** Formal analysis (equal); investigation (equal); methodology (equal). **Vishal Kulkarni:** Investigation (equal); methodology (equal); project administration (equal); resources (equal). **Sukhbir Singh Chopra:** Supervision (equal); validation (equal); visualization (equal).

## FUNDING INFORMATION

There is no funding support received in any form for this letter to editor.

## CONFLICT OF INTEREST STATEMENT

None of the authors have potential or actual conflicts of interest.
